# Synthesis of Ultrahigh Molecular Weight Poly(methyl Methacrylate) via the Polymerization of MMA Initiated by the Combination of Palladium Carboxylates with Thiols

**DOI:** 10.3390/polym15112501

**Published:** 2023-05-29

**Authors:** Panpan Zhang, Qiongqiong Xu, Wenyu Mao, Jiaxing Lv, Haodong Tang, Huadong Tang

**Affiliations:** Institute of Industrial Catalysis, College of Chemical Engineering, Zhejiang University of Technology, Hangzhou 310014, China; zhangpanpan@zjut.edu.cn (P.Z.); a15239905115@163.com (Q.X.); 18357654972@163.com (W.M.); 18846056271@163.com (J.L.); tanghd@zjut.edu.cn (H.T.)

**Keywords:** ultrahigh molecular weight, PMMA, polymerization, kinetics, mechanism

## Abstract

A novel synthesis of ultrahigh molecular weight poly(methyl methacrylate) (PMMA) using organosulfur compounds combined with a catalytical amount of transition metal carboxylates as an initiator has been developed. The combination of 1-octanethiol with palladium trifluoroacetate (Pd(CF_3_COO)_2_) was found to be a very efficient initiator for the polymerization of methyl methacrylate (MMA). An ultrahigh molecular weight PMMA with a number-average molecular weight of 1.68 × 10^6^ Da and a weight-average molecular weight of 5.38 × 10^6^ Da has been synthesized at the optimal formulation of [MMA]:[Pd(CF_3_COO)_2_]:[1-octanethiol] = 94,300:8:23 at 70 °C. A kinetic study showed that the reaction orders with respect to Pd(CF_3_COO)_2_, 1-octanethiol, and MMA are 0.64, 1.26, and 1.46, respectively. A variety of techniques such as proton nuclear magnetic resonance spectroscopy (^1^H NMR), electrospray ionization mass spectroscopy (ESI-MS), size exclusion chromatography (SEC), X-ray photoelectron spectroscopy (XPS), transmission electron microscopy (TEM), and electron paramagnetic resonance spectroscopy (EPR) were employed to characterize the produced PMMA and palladium nanoparticles (Pd NPs). The results revealed that Pd(CF_3_COO)_2_ was firstly reduced by the excess of 1-octanethiol to form Pd NPs at the early stage of the polymerization, followed by the adsorption of 1-octanethiol on the surface of nanoparticles and subsequent generation of corresponding thiyl radicals to initiate the polymerization of MMA.

## 1. Introduction

Poly(methyl methacrylate) (PMMA), one of the best colorless and transparent thermoplastic resins, is obtained via the polymerization of methyl methacrylate (MMA). PMMA has excellent comprehensive properties such as strong mechanical strength, clear light transmission, superior electrical insulation, extraordinary weather resistance, good heat resistance, etc. [1,2]. Therefore, PMMA has been widely used in optical lighting, furniture decorations, biomedical materials, sanitary wares, and many other industries [3,4,5,6,7]. However, common PMMA shows insufficient surface hardness and poor resistance to organic solvents, as well as low abrasion resistance [8], and is prone to stress cracking when subjected to heavy loads for a long time.

The mechanical properties of polymers have long been known to be dependent on their molecular weights. For example, the tensile strength, impact resistance, abrasion resistance, and weather resistance of PMMA are enhanced when increasing PMMA molecular weights [9,10,11]. Cooper and coworkers reported that, for stretched PMMA film with a broad molecular weight distribution, its maximum attainable tensile strength increased when the molecular weight increased up to 2.5 × 10^6^ Da, while the maximum tensile strength for fractionated PMMA sample increased linearly with molecular weight up to about 1.0 × 10^6^ Da and began to level off at higher molecular weight regions [12,13]. As the application demand for PMMA in new industrial fields (e.g., 5G communication, liquid crystal displays, new energy batteries, etc.) is increasing tremendously in recent years [14,15,16], it is of great significance to explore and develop new a synthetic approach at mild conditions for ultrahigh molecular weight PMMA.

Low-temperature suspension polymerization and low-temperature emulsion polymerization have conventionally been used to synthesize PMMA with molecular weights up to 10^6^ Da [17,18,19]. However, the post-treatment process after suspension or emulsion polymerization often generates a large amount of wastewater that contains suspension agents or emulsifiers, and it is difficult to completely remove these residual emulsifiers and suspension agents in final PMMA products, leading to the deterioration of their optical and electronic properties such as insufficient light transparency, weak insulation resistance, and low heat resistance.

Osada et al. reported that low-pressure plasma could initiate the polymerization of MMA and obtained ultrahigh molecular weight PMMA with a viscosity-average molecular weight higher than 10^7^ Da [20,21,22,23,24]. Li et al. also prepared PMMA polymers with viscosity-average molecular weight as high as 1.1 × 10^7^ Da by plasma-initiated emulsion polymerization [25]. Nevertheless, the plasma-initiated polymerization faced many challenges, such as slow polymerization rate, low production efficiency, demand for high power radiofrequency generator, and scale-up difficulties [26,27].

Lewis pair-catalyzed living polymerization using organoaluminum as a corresponding Lewis acid has been recently applied to prepare ultrahigh molecular weight PMMA with a number-average molecular weight of up to 1.927 × 10^6^ Da and narrow molecular weight distribution [28]. However, as the catalyst loading in the synthesis was relatively high and the organoaluminum compound was moist and air-sensitive, the polymerization increased technical difficulties in industrial applications. Reversible addition-fragmentation chain transfer (RAFT) polymerization [29,30], atom transfer radical polymerization (ATRP) [31,32,33,34,35,36,37], nitroxide-mediated polymerization (NMP) [38,39], and many other ‘‘living’’/controlled radical polymerization have also been adopted to polymerize MMA. For example, ultrahigh molecular weight PMMA with a number-average molecular weight of 3.60 × 10^6^ Da and 1.25 × 10^6^ Da have been prepared at very high pressure conditions (5000 bar) by ATRP and RAFT [40,41], respectively. Inevitably, such a high pressure causes extra production risks and high scale-up costs.

We have previously demonstrated that transition metal nanoparticles, especially the nearly monodispersed palladium nanoparticles (Pd NPs) combined with organic halides, could initiate the polymerization of MMA and produce ultrahigh molecular weight PMMA via a free radical mechanism [42]. As monodispersed noble metal nanoparticles are expensive and prone to aggregation in solution during storage, in this work, a series of organosulfur compounds combined with a catalytical amount of transition metal carboxylates were explored, and the combination of 1-octanethiol with palladium trifluoroacetate (Pd(CF_3_COO)_2_) was found to be very efficient to synthesize ultrahigh molecular weight PMMA under mild conditions. The kinetics of the polymerization have been investigated, and a variety of characterization techniques such as proton nuclear magnetic resonance (^1^H NMR) spectroscopy, transmission electron microscopy (TEM), size exclusion chromatography (SEC), electrospray ionization mass spectroscopy (ESI-MS), X-ray photoelectron spectroscopy (XPS), and electron paramagnetic resonance (EPR) have been used to characterize PMMA and reveal the polymerization mechanism.

## 2. Materials and Methods

### 2.1. Materials

Methyl methacrylate (MMA, 99.5%), Pd(CF_3_COO)_2_ (98.0%), palladium acetate (≥98.0%), iron stearate (98.0%), cobalt isocaprylate (98.0%), copper acetate (>98.0%), silver trifluoroacetate (98.0%), 1-octanethiol (>95%), dibutyl disulfide (99.0%), 4,4′-dinitrodibutyl disulfide (95.0%), and deuterated chloroform (99.9%) were purchased from Shanghai Aladdin Biochemical Technology Co. (Shanghai, China). *N*-tert-butyl-alpha-phenylnitrone (PBN, >98%) and 1,1-diphenyl-2-trinitrophenylhydrazine (DPPH, 97%) were supplied by TCI chemicals (Shanghai, China). Acetone (AR grade), methanol (AR grade), toluene (AR grade), tetrahydrofuran (THF, AR grade), hexane (AR grade), basic aluminum trioxide (200–300 mesh), molecular sieve (3Å, 2–3 mm), and all other chemicals were provided by Tianjin Yongda Chemical Reagent Co. (Tianjin, China).

The inhibitors in MMA were removed by passing through a column filled with basic aluminum oxide (200–300 mesh), and the purified monomer was dried using a molecular sieve (3Å, 2–3 mm) and stored at −20 °C in a refrigerator.

### 2.2. Synthesis of PMMA Using Organosulfur Compounds Combined with Transition Metal Carboxylates as an Initiator

A general procedure for the synthesis of PMMA using the combination of organosulfur compounds with a catalytical amount of transition metal carboxylates as an initiator is as follows: A certain amount of transition metal carboxylate (e.g., Pd(CF_3_COO)_2_, 3.3 mg) was weighed and added to a Schlenk reaction tube charged with a magnetic stirrer. The tube was sealed with a rubber septum and then subjected to high vacuum–nitrogen purge cycles. MMA (25.0 mL) was purged with nitrogen and added into the reaction tube by using a syringe under the protection of nitrogen, followed by the addition of organosulfur compound (e.g., 1-octanethiol, 12.0 μL) using a microsyringe. The reaction tube was subsequently transferred to a heating plate set at our desired temperature. At different time intervals, a long-needle syringe was purged with nitrogen and used to withdraw the reaction mixture. The obtained samples were put in vials and stored in a fridge for subsequent ^1^H NMR measurement, SEC analysis, and monomer conversion determination.

### 2.3. Characterization

Monomer conversion was generally measured using a gravimetric method. The ^1^H NMR spectrum of PMMA was acquired using a Bruker Avance III 500 MHz spectrometer with chloroform-d (CDCl_3_) as a deuterated solvent and tetramethylsilane (TMS, δ = 0 ppm) as an internal standard. To facilitate the end-group analysis of synthesized PMMA by ^1^H NMR, a low molecular weight PMMA was prepared so that the end groups could occupy relatively high content in the polymer. The procedure to prepare low molecular weight PMMA is the same as the above general synthetic steps of PMMA, except the reaction needs to be stopped at a low monomer conversion (~5%). The low-conversion reaction solution was precipitated in excessive cold hexane, and the obtained low molecular weight PMMA precipitates were dried under vacuum.

The number-average molecular weight (*M*_n_), weight-average molecular weight (*M*_w_), and the dispersity index (Đ, Đ = *M*_w_/*M*_n_) of synthesized PMMA were determined by a Shimadzu SEC system (Shimadzu Co., Kyoto, Japan) equipped with a Shimadzu liquid transfer unit LC-16, a Shimadzu refractometer RID-20A, a Shimadzu column oven CTO-16, and a Waters solvent-efficient Styragel HR-5E high-resolution column (4.6 mm × 300 mm, molecular weight range: 2.0 × 10^3^ Da–4.0 × 10^6^ Da). THF was employed as an eluent with a flow rate of 0.3 mL/min at 35 °C. Data were collected and processed using Labsolution software (Ver. 5.82, Shimadzu Co., Kyoto, Japan). A series of PMMA standards (Polymer Laboratories, Lewiston, ME, USA, molecular weight range: 1020 Da–1,944,000 Da) were used to generate the SEC calibration curve.

The morphology of in situ-formed Pd NPs in the polymerization of MMA was observed by using a Tecnai G2 F30 transmission electron microscope (TEM, acceleration voltage 300 kV, FEI, Eindhoven, Netherlands). This microscope is equipped with an energy-dispersive X-ray spectrometer (EDX) and has a point resolution of 0.20 nm and a line resolution of 0.10 nm. To collect the in situ-formed Pd NPs, the polymerization of MMA initiated by the combination of 1-octanethiol with (Pd(CF_3_COO)_2_) was stopped at the early stage of the reaction (~5% conversion, 1.5 h~2 h) to avoid separation difficulties at high conversion due to polymer viscosity. A total of 1 mL of the reaction solution was mixed with 1 mL of THF and then centrifuged at 12,000 rpm for 20 min. The upper solution was removed by using a dropper, and the bottom droplet was redispersed in 200 μL THF under ultrasonication. The THF solution was further dripped on a TEM copper grid and left to dry in the air before TEM analysis took place.

To collect enough in situ-produced Pd NPs for XPS analysis, the above polymerization was stopped at a low monomer conversion (~5%, 1.5 h~2 h), and then the whole reaction solution was diluted in an equal volume of THF. The mixture was centrifuged at 12,000 rpm for 20 min, and the bottom droplet was redispersed in 5 mL of THF under ultrasonication. Finally, the THF solution was centrifuged again at 12,000 rpm for 20 min, and the bottom pellet was dried under vacuum at room temperature and collected as a black powder. This procedure was repeated to accumulate enough Pd NPs for XPS analysis. Generally, 4.0 mg of collected Pd NPs were spread on the surface of a silicon wafer (8 mm × 8 mm), and the XPS measurement was performed using a Kratos AXIS Ultra DLD spectrometer with a monochromatic Al target X-ray source. The pressures of sample analysis and processing chamber were below 5 × 10^−10^ and 5 × 10^−9^ Torr, respectively.

The exact molecular weight of prepared low molecular weight PMMA was determined using a LCQ Deca XP (Thermo Electron Corporation, Waltham, MA, USA) electrospray ionization mass spectrometer (ESI-MS). The instrument was operated in a positive ion mode with a capillary temperature of 350 °C, a spray voltage of 4.5 kV, and an ion gauge pressure of 2.4 × 10^−5^ Torr. The low-molecular-weight PMMA sample was dissolved in acetone (~5 wt%) and diluted in methanol. A sample infusion flow rate of 10 μL/min was employed.

EPR technique was used to identify radical species in the polymerization of MMA. The measurement was carried out using a Bruker A300 electron paramagnetic resonance spectrometer. The instrument worked in X-band with a microwave power of 19.9 mw at a frequency of 9.4177 GHz, and was calibrated using DPPH as a standard. PBN was added into the reaction solution during the initial stage of the polymerization of MMA (PBN concentration of 0.075 mol/L) to capture any radical intermediates or species. A total of 50 μL of the mixture solution was sealed into a 1.3 mm quartz capillary tube under the protection of nitrogen for EPR measurement.

## 3. Results and Discussion

### 3.1. Synthesis of Ultrahigh Molecular Weight PMMA Using the Combination of Organosulfur Compounds with Transition Metal Carboxylates as an Initiator

In previous work, we have reported that certain organic halides combined with Pd NPs could initiate the polymerization of many vinyl monomers and produce ultrahigh molecular weight PMMA. However, monodispersed or nearly monodispersed noble metal NPs are generally expensive. The synthesis and purification processes of nearly monodispersed Pd NPs are tedious, time-consuming, and cost-ineffective. In addition, Pd NPs are prone to aggregation in solution during long time storage, which may cause significant application difficulty at industrial scales. Therefore, in this research, we tried to replace the nearly monodispersed Pd NPs with transition metal carboxylates and found that certain combinations of transition metal carboxylates with organosulfur compounds could initiate the polymerization of MMA and generate ultrahigh molecular weight PMMA, as listed in Table 1.

Obviously, the transition metal carboxylate alone (e.g., Pd(CF_3_COO)_2_, Table 1, entry 1) or the organosulfur compound itself, such as 1-octanethio or dibutyl disulfide (entry 2, 3), could not initiate the polymerization of MMA under the present study’s experimental conditions. Even in the case where dibutyl disulfide was combined with palladium, cobalt, or copper carboxylate, it still could not start the polymerization of MMA (entry 4–6). In comparison with dibutyl disulfide, 4,4′-dinitrodiphenyl disulfide combined with silver or Pd(CF_3_COO)_2_ (entry 7, 8) promoted the polymerization of MMA, but the conversion was low, implying that the electron-withdrawing groups attached to benzene might activate the disulfide linkage in 4,4′-dinitrodiphenyl disulfide.

**Figure 1:**
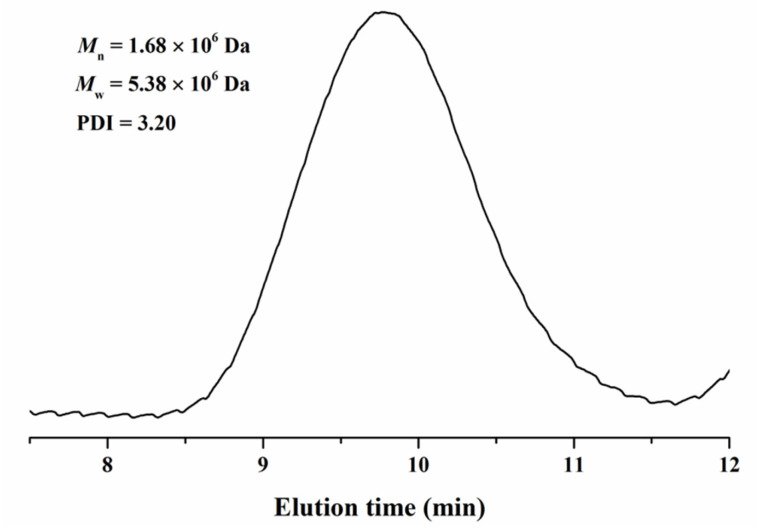
SEC curve of the ultrahigh molecular weight PMMA synthesized by the polymerization of MMA using the combination of 1-octanethiol with Pd(CF_3_COO)_2_ as an initiator.

Thiols are generally recognized as chain transfer agents and often used in free radical polymerizations to control polymer molecular weights. Interestingly, in this work, a trace amount of 1-octanethiol or 1-butanethiol (less than 0.05 wt%) combined with catalytical amount of copper, silver, iron, or palladium carboxylate was found to facilitate the polymerization of MMA (entry 9–15). Specifically, the combination of 1-octanethiol with Pd(CF_3_COO)_2_ at the optimal formulation ([MMA]:[Pd(CF_3_COO)_2_]:[1-octanethiol] = 94,300:8:23, entry 15) accelerated the polymerization of MMA to a 92.60% conversion in 48 h and generated an ultrahigh molecular weight PMMA with a number-average molecular weight of 1.68 × 10^6^ Da and a weight-average molecular weight of 5.38 × 10^6^ Da, as measured by SEC (Figure 1), suggesting that these thiols played the role of an initiator rather than a chain transfer agent in the polymerization since their concentration was extremely low, and the combination of 1-octanethiol with Pd(CF_3_COO)_2_ could be employed as an efficient initiator for the polymerization of MMA to synthesize ultrahigh molecular weight PMMA.

### 3.2. Kinetics of the Polymerization Initiated by the Combination of 1-Octanethiol with Pd(CF_3_COO)_2_

**Figure 2:**
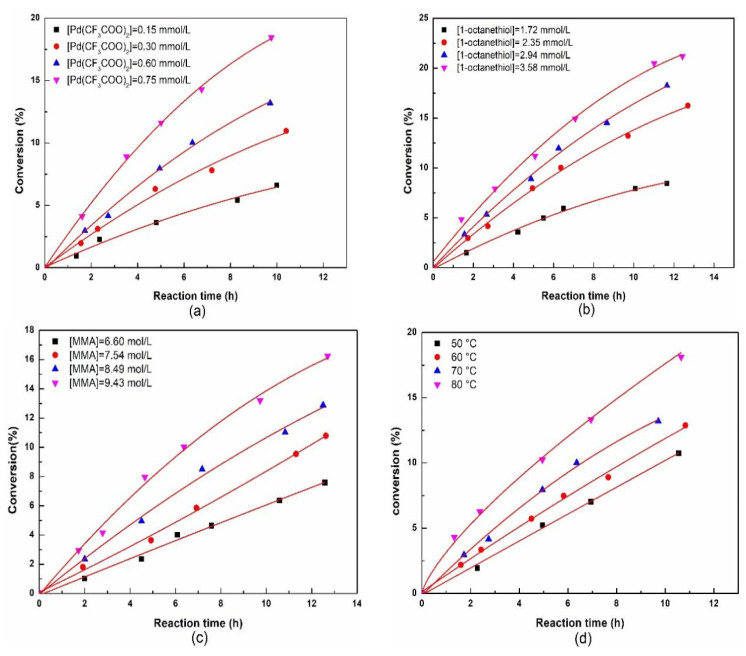
Kinetic plots for the polymerization of MMA initiated by the combination of 1-octanethiol with Pd(CF_3_COO)_2_ at different Pd(CF_3_COO)_2_ concentrations (**a**), different 1-octanethiol concentrations (**b**), different MMA concentrations (**c**), and different temperatures (**d**).

The polymerization kinetics initiated by the combination of 1-octanethiol with Pd(CF_3_COO)_2_ based on the optimal formulation was investigated, and the results are shown in Figure 2.

Clearly, the rate of the polymerization increased with the increase in Pd(CF_3_COO)_2_ concentration, 1-octanethiol concentration, and MMA concentration, and also increased with the increase in temperature. The reaction orders with respect to Pd(CF_3_COO)_2_, 1-octanethiol, and MMA were calculated to be 0.64, 1.26, and 1.46, respectively, according to the initial reaction rates measured at the initial stage of the polymerization (conversion < 10%) in Figure 2a–c; therefore, the polymerization kinetic equation is given as *R* = -d[M]/dt = *k*[Pd(CF_3_COO)_2_]^0.64^[1-octanethiol]^1.26^[MMA]^1.46^. Based on Figure 2d, the apparent activation energy *E*_a_ of the polymerization was determined to be 40.0 kJ/mol, according to Arrhenius equation.

It has been well documented that the reaction orders with respect to the initiators and monomers in traditional free radical polymerization are 0.5 and 1, respectively; therefore, the above kinetic results imply that the polymerization of MMA initiated by the combination of 1-octanethiol with a catalytical amount of Pd(CF_3_COO)_2_ is not a typical conventional free radical polymerization.

### 3.3. Preliminary Investigation of the Mechanism of MMA Polymerization Initiated by the Combination of 1-Octanethiol with Pd(CF_3_COO)_2_

#### 3.3.1. TEM Images and XPS Analysis of the Nanoparticles In Situ Formed at the Early Stage of the Polymerization

**Figure 3:**
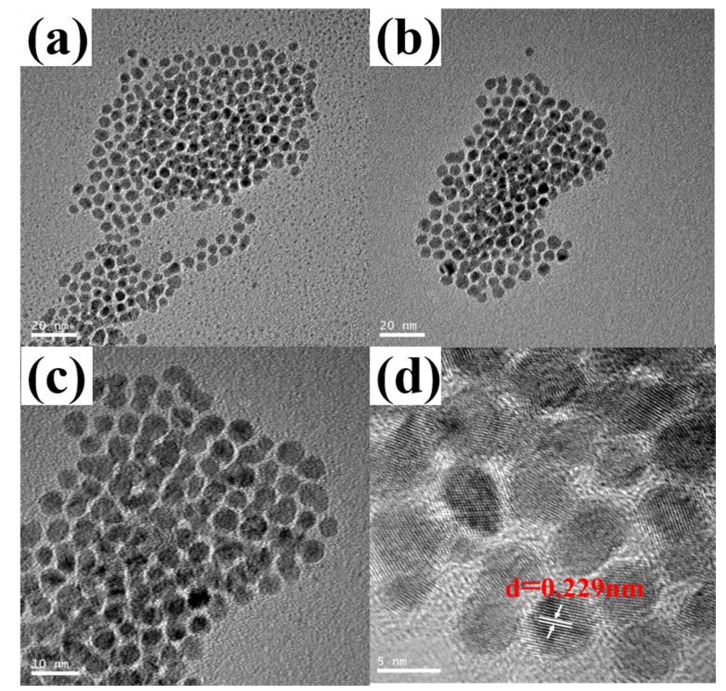
Normal-resolution TEM images (**a**–**c**) and high-resolution image (**d**) of the in situ-formed nanoparticles at the early stage of the polymerization initiated by the combination of 1-octanethiol with Pd(CF_3_COO)_2_.

As the polymerization of MMA initiated by the combination of 1-octanethiol with Pd(CF_3_COO)_2_ did not follow a traditional free radical polymerization mechanism, it is necessary to investigate the nature of the polymerization reaction. Inspired by our previous research in which Pd nanoparticles functioned as a catalyst to promote the formation of organic radicals [42], it is presumed in this work that the reductive thiol may reduce the oxidative Pd^2+^ to form zero-valent Pd NPs. This presumption was confirmed by the TEM results, as shown in Figure 3.

Figure 3 presents normal- (a–c) and high-resolution (d) TEM images of the particle samples collected from the reaction solution at the early stage (~5% conversion, 1.5 h~2 h) of the polymerization of MMA. Obviously, a lot of nanoparticles with a size of about 2 nm~6 nm were observed. The average particle diameter of these nanoparticles was determined to be 3.50 ± 0.43 nm using Nanomeasure software (Ver 1.2). The high-resolution image shows clear crystal lattice fringes, and the average distance between the two neighboring fringes was measured to be 0.229 nm, corresponding to the (111) plane of palladium crystals [43].

**Figure 4:**
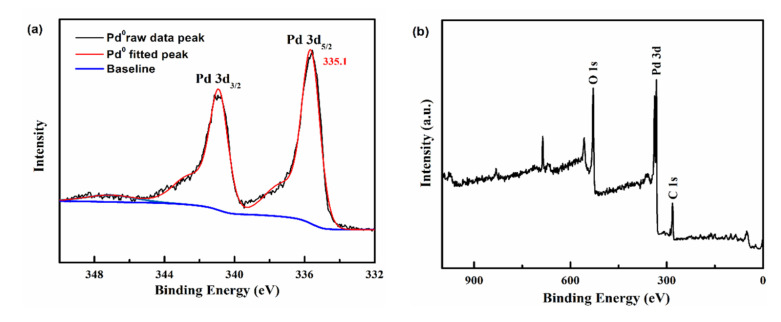
XPS photoelectron spectra of in situ-formed Pd NPs at the early stage of the polymerization of MMA; (**a**) XPS spectrum of Pd 3d, and (**b**) wide-scan spectrum of the Pd NPs.

These Pd NPs were further confirmed by XPS analysis, as shown in Figure 4. All binding energy peaks in XPS spectra were calibrated by C1s peak at 284.6 eV, as shown in Figure 4b. According to the literature, the two binding energy peaks are ascribed to Pd^0^ 3d_3/2_ and Pd^0^ 3d_5/2_ (Figure 4a) [44], proving that the tested nanopowders are zero-valent Pd NPs.

#### 3.3.2. EPR Analysis for the Polymerization Initiated by the Combination of 1-Octanethiol with Pd(CF_3_COO)_2_

**Figure 5:**
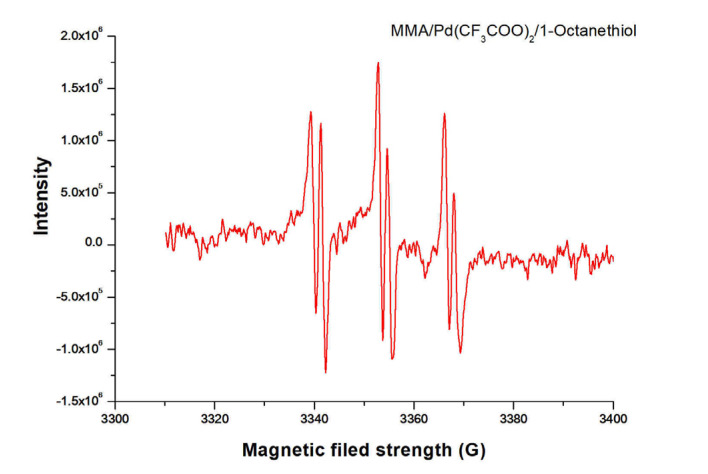
EPR spectrum recorded from the polymerization solution initiated by the combination of 1-octanethiol with Pd(CF_3_COO)_2_ using PBN as a radical trapping agent (PBN concentration: 0.075 mol/L).

The above results demonstrated that Pd(CF_3_COO)_2_ was indeed reduced by 1-octanethiol to Pd NPs in the early stage of the polymerization, and more importantly, the results implied that the polymerization of MMA initiated by the combination of 1-octanethiol with Pd(CF_3_COO)_2_ might also follow a radical mechanism, as proposed in our previous work [42]. EPR technique was hence employed to identify active radical species in the polymerization. Figure 5 shows the EPR spectrum recorded from the polymerization solution using PBN as a radical trapping agent.

Clearly, three sets of sixfold peaks were observed. According to the literature [45,46,47,48], the triple doublet signals are the result of the hyperfine coupling of carbon radicals to the ^14^N nucleus and β-proton of PBN, indicating that the active center of polymerization chain growth is a carbon radical, thus further affirming that the 1-octanethiol/Pd(CF_3_COO)_2_ initiated polymerization of MMA underwent a radical polymerization process.

#### 3.3.3. End-Group Analysis of the Prepared PMMA Macromolecular Chains

**Figure 6:**
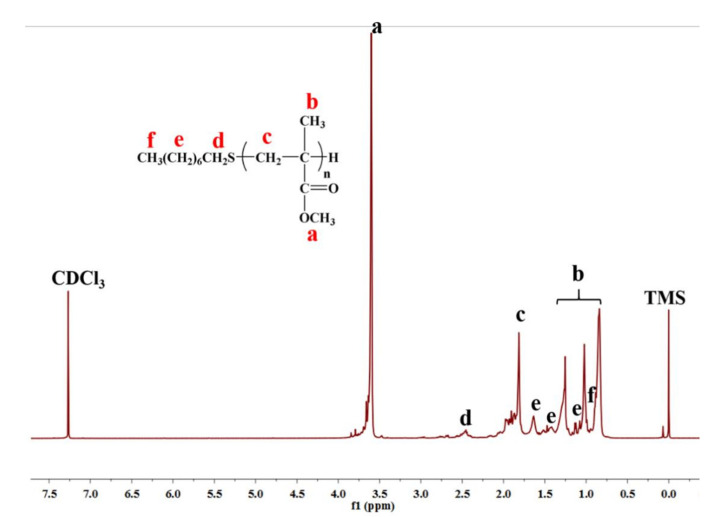
^1^H NMR spectrum of prepared low-molecular-weight PMMA.

As we have proved that the polymerization initiated by the combination of 1-octanethiol with a catalytical amount of Pd(CF_3_COO)_2_ followed a radical mechanism, it is important to study the polymer chain structures and figure out the end groups of macromolecular chains. A low-molecular-weight PMMA was prepared and its number-average molecular weight M_n_ was determined to be 1310 Da by SEC. The ^1^H NMR spectrum of the low molecular weight PMMA is shown in Figure 6.

As can be seen in Figure 6, besides the internal standard TMS at δ = 0 ppm and solvent CDCl_3_ peak at δ = 7.27 ppm, all the other peaks are ascribed to the polymer PMMA. According to the literature [49,50], the strong peak **a** (δ = 3.6 ppm) is the proton resonance of the methoxy group (-OCH_3_) of PMMA; the triple peak **b** (δ = 0.8, 1.0, 1.25 ppm) corresponds to methyl peak (-CH_3_) of PMMA and is similar to the triple peak of PMMA prepared by conventional free radical polymerization [50], implying that the PMMA primarily consists of syndiotactic conformation. Peak **c** (δ = 1.8~2.0 ppm) is the signal from the methylene group (-CH_2_-) of PMMA. Since we used 1-octanethiol as an initiator, 1-octanethiol residue could be reasonably assumed to attach at the end of the PMMA chain, as illustrated in the inset structure in Figure 6. Therefore, the tiny peak **d** (δ = 2.5 ppm) can be assigned to the methylene group [CH_3_(CH_2_)_6_**CH**_2_S-] immediately adjacent to the sulfide atom of 1-octanethiol residue, and the other methylene groups in 1-octanethiol residue [CH_3_(**CH**_2_)_6_CH_2_S-] show resonances at peak **e** (δ = 1.1~1.6 ppm). The methyl peak of 1-octanethiol residue [**CH**_3_(CH_2_)_6_CH_2_S-] is supposed to overlap with the methyl peak of the PMMA chain. According to the peak integration areas of **a** and d, the molecular mass of PMMA is thus calculated to be 1045 Da, which is close to the molecular weight measured by SEC.

**Figure 7:**
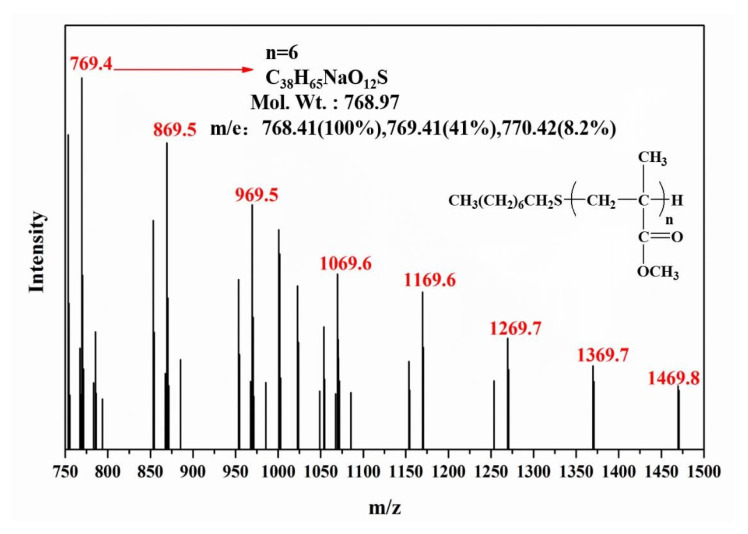
Electrospray ionization mass spectrum of prepared low-molecular-weight PMMA.

The end-group structure of PMMA was further verified by ESI-MS, as shown in Figure 7. A series of homologous molecular ion peaks (*m*/*z* = 769.4, 869.5, 969.5, 1069.6, 1169.6, 1269.7, 1369.7, 1469.8) can be observed, and the mass difference between two neighboring major peaks remains 100, which corresponds to the molecular weight of PMMA structural unit (C_5_H_8_O_2_). The peak, for example, at *m*/*z* = 769.4 is consistent with the Na^+^ adduct of the PMMA macromolecular chain with six structural units and an initiator (1-octanethiol), indicating that the end-group structure proposed in the inset in Figure 6 is correct.

### 3.4. Postulated Mechanism for the Polymerization of MMA Initiated by the Combination of 1-Octanethiol with Pd(CF_3_COO)_2_

**Figure 8:**
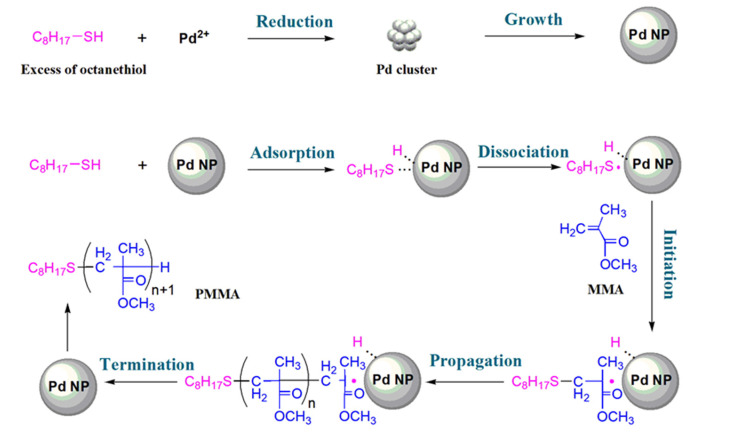
A postulated mechanism for the polymerization of MMA initiated by the combination of 1-octanethiol with Pd(CF_3_COO)_2_.

Based on the above experimental results including the following kinetics: TEM, XPS, ^1^H NMR, EPR, and ESI-MS, a plausible mechanism is postulated as follows (Figure 8).

Pd(CF_3_COO)_2_ is firstly reduced by 1-octanethiol to produce Pd clusters and further grow to Pd NPs at the early stage of the polymerization, as there is a higher abundance of 1-octanethiol compared to Pd(CF_3_COO)_2_. 1-Octanethiol is then adsorbed on the surface of Pd NPs to form a C_8_H_17_S-Pd bond, which is activated at relatively high temperatures in the polymerization conditions to release thiyl radicals around Pd NPs. The thiyl radical could immediately initiate MMA monomer [51] around the surface of nanoparticles, followed by continuous chain propagation of the polymerization. As the reaction primarily occurs in the vicinity of Pd NPs, these nanoparticles are assumed to provide certain protections to the propagating radicals and somewhat suppress the radical bimolecular terminations due to their giant size as physical barriers in comparison to macromolecular chains. So, the Pd(CF_3_COO)_2_/1-octanethiol-initiated polymerization of MMA in this work may not comply with a traditional free radical polymerization mechanism. Radical bimolecular terminations in this system are supposed to be restrained, consequently facilitating the synthesis of ultrahigh molecular weight PMMA.

## 4. Conclusions

In this work, a novel synthesis of ultrahigh molecular weight PMMA using organosulfur compounds combined with a catalytical amount of transition metal carboxylates as an initiator has been developed. The bulk polymerization of MMA initiated by the combination of 1-octanethiol with Pd(CF_3_COO)_2_ at the optimal formulation of [MMA]:[Pd(CF_3_COO)_2_]:[1-octanethiol] = 94,300:8:23 reached 92.6% conversion in 48 h at 70 °C in the atmosphere of N_2_, producing an ultrahigh molecular weight PMMA with a number-average molecular weight of 1.68 × 10^6^ Da, a weight-average molecular weight of 5.38 × 10^6^ Da, and a Đ of 3.20. The reaction orders with respect to Pd(CF_3_COO)_2_, 1-octanethiol, and MMA were determined to be 0.64, 1.26, and 1.46, respectively; thus, the polymerization rate *R* could be written as *R* = -d[M]/dt = *k*[Pd(CF_3_COO)_2_]^0.64^[1-octanethiol]^1.26^[MMA]^1.46^ (*k* is the reaction rate constant). A mechanism investigation revealed that Pd(CF_3_COO)_2_ is firstly reduced by 1-octanethiol to form Pd NPs in situ, followed by the adsorption of 1-octanethiol on the surface of nanoparticles and subsequent release of thiyl radicals to initiate the polymerization of MMA.

## Figures and Tables

**Table 1:** Polymerization of MMA initiated by the combination of organosulfur compounds with transition metal carboxylates ^1^.

Entry	Transition Metal Carboxylate	Organosulfur Compound	[MMA]: [M]:[S] ^2^	Time (h)	Conv. (%)	*M*_n_ (Da)	*M*_w_ (Da)	Đ
1	Pd(CF_3_COO)_2_	-	94,300:4:0	12	-	-	-	-
2	-	1-octanethiol	94,300:0:28	12	-	-	-	-
3	-	dibutyl disulfide	94,300:0:28	12	-	-	-	-
4	Pd(CF_3_COO)_2_	dibutyl disulfide	94,300:4:28	12	-	-	-	-
5	cobalt isocaprylate	dibutyl disulfide	94,300:4:28	12	-	-	-	-
6	copper acetate	dibutyl disulfide	94,300:4:28	12	-	-	-	-
7	silver trifluoroacetate	4,4′-dinitrodiphenyl disulfide	94,300:4:28	12	10.26	9.78 × 10^4^	3.41 × 10^5^	3.49
8	Pd(CF_3_COO)_2_	4,4′-dinitrodiphenyl disulfide	94,300:4:28	12	23.26	1.58 × 10^5^	4.42 × 10^5^	2.80
9	copper acetate	1-octanethiol	94,300:4:28	12	8.59	8.59 × 10^4^	3.66 × 10^5^	4.26
10	iron stearate	1-octanethiol	94,300:4:28	12	7.43	9.57 × 10^4^	3.43 × 10^5^	3.58
11	silver trifluoroacetate	1-octanethiol	94,300:4:28	12	2.55	2.96 × 10^5^	9.75 × 10^5^	3.29
12	Pd(CF_3_COO)_2_	1-butanethiol	94,300:4:28	12	42.33	7.44 × 10^5^	2.71 ×10^6^	3.64
13	Pd(CF_3_COO)_2_	1-octanethiol	94,300:4:28	12	62.54	1.12 × 10^6^	4.07 × 10^6^	3.64
14	Pd(CF_3_COO)_2_	1-octanethiol	94,300:8:23	36	76.52	1.35 × 10^6^	4.81 × 10^6^	3.56
15	Pd(CF_3_COO)_2_	1-octanethiol	94,300:8:23	48	92.60	1.68 × 10^6^	5.38 × 10^6^	3.20

^1^ All polymerizations were conducted at 70 °C under the protection of nitrogen. ^2^ [MMA]:[M]:[S] = [monomer]:[transition metal carboxylate]:[organosulfur compound].

## Data Availability

Not applicable.
